# Monitoring House Fly (Diptera: Muscidae) Activity on Animal Facilities

**DOI:** 10.1093/jisesa/ieaa109

**Published:** 2020-11-02

**Authors:** Alec C Gerry

**Affiliations:** Department of Entomology, University of California at Riverside, Riverside, CA

**Keywords:** surveillance, nuisance, IPM, abundance, CAFO

## Abstract

Monitoring house fly (Diptera: Muscidae) activity on animal facilities is a necessary component of an integrated pest management (IPM) program to reduce the negative impacts of these flies. This article describes monitoring methods appropriate for use on animal facilities with discussion of monitoring device use and placement. Action thresholds are presented where these have been suggested by researchers. Sampling precision is an important aspect of a monitoring program, and the number of monitoring devices needed to detect a doubling of fly activity is presented for monitoring methods where this information is available. It should be noted that both action thresholds and numbers of monitoring devices will be different for every animal facility. Suggested action thresholds and numbers of monitoring devices are presented only to provide guidance when initiating a fly monitoring program. Facility managers can adjust these values based upon the fly activity data recorded at their facility. Spot cards are generally recommended as an easy-to-use method for monitoring fly activity for most animal facilities. Fly ribbons or similar sticky devices are recommended where several pest fly species may be abundant and identifying the activity of each species is important, but a sampling period of <7 d may be needed in dusty conditions or when fly density is high. Fly ribbons are not recommended for outdoor use. Insecticide-baited traps may be used in outdoor locations where environmental conditions limit the use of spot cards, fly ribbons, and sticky traps.

The house fly (*Musca domestica* L.) (Diptera: Muscidae) is a ubiquitous pest often associated with animal production facilities where these flies develop in the animal feces and decaying organic matter that is often abundant at these facilities (Geden and Hogsette 1994). When large numbers of house flies are produced, flies can cause nuisance to facility workers and to the surrounding community potentially resulting in citations, fines, and even lawsuits (Thomas and Skoda 1993, Winpisinger et al. 2005). House flies can also transmit several animal and human pathogens (Greenberg 1971, Olsen 1998, Nayduch and Burrus 2017). Control of house flies produced on and dispersing from animal facilities is important to reduce the potential for these negative impacts.

To reduce house fly nuisance and the potential for pathogen transmission, a fly management program should generally follow integrated pest management (IPM) principles including ongoing monitoring of house fly activity so that fly control can be initiated when needed to keep fly activity below a level (‘action threshold’) where nuisance or pathogen transmission might be anticipated (Stern et al. 1959, Flint and van den Bosch 1981). Monitoring of house fly activity is also needed to confirm effectiveness of fly control efforts so that facility operators do not continue to use ineffective measures such as insecticides to which house flies have developed resistance (Keiding 1999, Freeman et al. 2019). Finally, a house fly monitoring program will provide a historical record of fly activity that can be useful for facility managers to address complaints levied against the facility.

There are many methods to monitor house fly abundance and activity (reviewed by Gerry 2020). Unfortunately, house fly monitoring methods have not been standardized for any animal production system. This lack of standardization in fly monitoring methods has limited implementation of comprehensive fly management programs rooted in the IPM concept leading to few animal facilities routinely monitoring fly activity as a key component of their control program unless required by federal or local regulations. For example, egg-layer facilities are required to monitor house fly activity as part of the U.S. Food and Drug Administration *Salmonella* Enteritidis prevention strategy (FDA 2009). Without a fly monitoring program, fly control is often initiated only when adult fly activity has resulted in negative impacts to the facility or to the surrounding community, suggesting that the action threshold was already exceeded and adult fly activity must be immediately reduced typically using insecticides targeting adult house flies (Geden and Hogsette 1994).

In this article, methods for monitoring house fly activity are recommended for various animal facilities. Methods include description of monitoring devices including number and placement of devices where published information is available to provide guidance by type of animal facility. Any recommendations provided in this article are intended only as guidelines to support the development and implementation of an effective fly management program. Recommendations are not intended to be requirements for animal producers, since facility managers must select the monitoring method and application of that method that is most suited for their facility based upon the design, management, and animal density at the facility as well as the climate, surrounding geography, and character of the surrounding community (e.g., tolerance to flies).

## Monitoring Methods—General Considerations

There are many methods that have been used to monitor house fly activity, and these can be categorized by type of monitoring device as well as by sampling period (reviewed by Gerry 2020). House fly monitoring methods do not measure true abundance (or true density) of flies at the sampled facility, as this requires more challenging techniques (Kristiansen and Skovmand 1985). Instead, fly monitoring methods provide a measure or index of house fly activity that is related to fly density at the sampled location and to the frequency of individual fly behaviors, such as flight, that alter the rate of fly contact with monitoring devices (Gerry 2020). The risk of nuisance and pathogen transmission by flies is likely also related to overall fly activity, including fly density and the frequency of fly behaviors (e.g., flight, feeding, response to attractive odors, deposition of fecal and regurgitation spots), though this has not been carefully evaluated. Since the purpose of a house fly monitoring program is largely to reduce nuisance and pathogen transmission, monitoring methods that measure fly activity (density and behavior) are likely to be most suitable.

Monitoring methods that record fly activity at a single point in time (‘instantaneous counts’), such as a visual count of flies resting at a particular location on the farm, have shown poor correlation to fly density (Pickens et al. 1972, Beck and Turner 1985) largely due to the impact of environmental conditions such as temperature on house fly activity, including flight behavior (Parker 1962, Zahn and Gerry 2020). Thus each fly activity count is greatly dependent upon the environmental conditions at the time the activity count is performed, and these conditions can vary considerably throughout the day so that a single time period count may not well represent fly activity during the rest of the day. Monitoring methods with a fly activity sampling period <24 h were also generally unsuitable for routine fly monitoring, perhaps due to similar impacts of environmental factors on fly activity recorded over a short period of time (Gerry 2020). Methods that accumulate flies on a monitoring device over a longer sampling period to ensure a range of environmental conditions experienced by the fly population being sampled are expected to provide a better estimate of overall fly activity (Lysyk and Moon 1994). Thus, only monitoring methods that record fly activity over ≥24 h are recommended below for routine monitoring of house fly activity on animal facilities.

House fly activity is usually performed on a weekly basis. For monitoring methods that are deployed for <7 d, the count may be recorded as a daily activity count (e.g., flies per day) rather than a weekly activity count. This is particularly important if the length of the monitoring period varies across weeks due to operational considerations. To minimize the impact of environmental conditions on the estimated house fly flight activity, it is important that monitoring devices are deployed and retrieved at approximately the same time of day each week. With fewer significant environmental impacts on flight activity during midday (Zahn and Gerry 2020), placement and retrieval of monitoring devices at a consistent midday time may be best.

The main goal for monitoring house fly activity is to ensure that fly activity remains at a level below the action threshold where negative impacts such as nuisance or pathogen transmission are anticipated to occur. As fly activity increases toward the action threshold, facility managers might consider implementing fly control measures, particularly those focused on reducing the production of adult flies, so that fly activity does not reach the action threshold (Urech et al. 2004). Above the action threshold, fly control efforts must immediately target adult flies, typically through use of insecticides (Geden and Hogsette 1994) to rapidly reduce fly activity by decreasing fly density.

Unfortunately, few action thresholds have been published for fly activity using any monitoring method. Also, where action thresholds have been published, these fly activity values were subjectively derived based upon researcher experience and are applicable only to the specific facilities and monitoring methods that were evaluated in the published studies. Published action thresholds are therefore not universal, but may provide a starting point for initiating a fly management program with improved action thresholds determined later based upon empirical analysis of fly activity records in the presence or absence of reported negative impacts by flies (e.g., nuisance complaints) (Gerry 2020).

Any monitoring method selected to routinely record fly activity must have enough sampling precision to distinguish a change in house fly activity, particularly when activity is near the action threshold. Sampling precision is a function of the mean and variance of fly activity data recorded by the monitoring devices, and is related to the density and distribution of flies and the design and number of monitoring devices used (Southwood 1978). Sampling precision typically increases as more monitoring devices are used, but since each monitoring device requires time to set up and process at the end of the sampling period, it is prudent to use only enough monitoring devices to provide a desired level of sampling precision. A monitoring method is sufficiently precise if it can detect a doubling of fly activity at the sampled location (Southwood 1978). Where numbers of sampling devices are indicated in the sections below, these are predicted to be sufficient to detect such a change in fly activity and may be used as general guidance for animal facilities of similar design. However, since sampling precision may differ even among very similar facilities using the same monitoring devices, a more accurate prediction of the minimum number of monitoring devices needed at a facility to achieve a suitable level of precision can be determined following methods outlined by Karandinos (1976) using house fly activity data recorded through the first seasonal peak in fly activity as suggested by Gerry (2020).

## Poultry Facilities

Methods for monitoring house fly activity at poultry facilities have been previously described for caged-layer houses with either a narrow or high-rise (deep-pit) house design (Anderson and Poorbaugh 1964; Axtell 1970a,b; Rutz and Axtell 1979; Burg and Axtell 1984; Beck and Turner 1985; Lysyk and Axtell 1986). Methods are more limited for broiler-breeder facilities due to dust accumulation negatively impacting monitoring devices that capture flies on a sticky surface (Rutz and Axtell 1981).

### Fly Ribbons

Sticky fly ribbons (also called fly tapes or fly papers) offer a simple method to assess adult fly activity with flies captured as they land to rest on the sticky surface of the fly ribbon. Fly ribbons take advantage of fly behavior to preferentially land on suspended or hanging objects (Howard 1911). Fly ribbons are relatively inexpensive and commercially available from many vendors. Each fly ribbon is usually supplied with a thumbtack to fasten the fly ribbon to hang freely from a wooden beam or similar structure, but for routine fly monitoring a screw hook purchased separately from a hardware store can be fixed to the wooden support for easy replacement of fly ribbons using the cloth loop at the top edge of the fly ribbon. Fly ribbons will capture several pest fly species that may be abundant at poultry facilities (Anderson and Poorbaugh 1964; Axtell 1970a,b; Legner et al. 1973; Lysyk and Axtell 1986) allowing each of these fly species to be monitored simultaneously.

Fly ribbons should be hung in protected locations where flies naturally gather (Kilpatrick and Quarterman 1952, Anderson and Poorbaugh 1964, Pickens et al. 1972). Within poultry houses, fly ribbons are typically hung from midline roof supports where they are reported to provide an effective measure of house fly activity (Anderson and Poorbaugh 1964; Axtell 1970a,b; Legner et al. 1973; Rutz and Axtell 1979; Quisenberry and Foster 1984; Lysyk and Axtell 1986; James et al. 2017). Fly ribbons may be ineffective in broiler-breeder houses due to dusty conditions resulting in fly ribbons losing their stickiness (Rutz and Axtell 1981). Placing fly ribbons in direct sunlight can also result in rapid loss of stickiness (Anderson and Poorbaugh 1965). Fly ribbons should be placed out of reach of birds and away from doorways or walkways where human activity can interfere with fly activity.

Fly ribbons hung from roof supports for ≥2 d provided fly activity counts that were generally related to fly density and to other measures of fly activity (Rutz and Axtell 1979; Lysyk and Axtell 1986) suggesting that this is an effective sampling period. While fly ribbons may also be hung for longer sampling periods, up to 7 d for efficiency of the monitoring program (Axtell 1970a,b; Lysyk and Axtell 1986), ribbons can fill with flies in just a few days when fly activity is high (Axtell 1970a) and length of the sampling period is therefore best determined by experience using fly ribbons during the peak fly season. Other sticky devices may be used similarly to fly ribbons, but there is little published information comparing fly ribbons to other devices. One sticky device that may show promise for routine house fly monitoring, at least in terms of ease of use, are sticky cards which can be readily applied to poultry housing support posts or similar locations (Hogsette et al. 1993, Geden et al. 1999).

Record weekly fly activity as the number of flies captured per ribbon per day (flies per ribbon per day) to compare fly activity across time even when there is variation in the sampling interval (# days) or the number of fly ribbons recovered at the end of the week. Suggested house fly activity action thresholds using fly ribbons hung from roof rafters in egg-layer houses range from 14–43 house flies per ribbon per day (Axtell 1970a,b; Lysyk and Axtell 1986). To detect a doubling of fly activity at the lowest suggested action threshold, a minimum of four or nine fly ribbons are needed (per narrow house or high-rise house, respectively) (Lysyk and Axtell 1986).

### Spot Cards

Spot cards (or fly speck cards) are white index cards pinned to locations where house flies are noted to deposit fecal and regurgitation spots (‘fly spots’), with the number of spots recorded as a relative measure of house fly activity (Axtell 1970a, 1986). Since fly spots are recorded, not flies, no identification skills are required by the facility operators. However, several pest fly species can deposit similar fly spots on the white cards, so spot counts are a measure of overall pest fly activity where flies other than house fly are present (Lysyk and Axtell 1986). Any size white index card will work as a spot card, though 3 × 5 inch (7.62 × 12.7 cm) cards are most commonly used. If cards of different sizes are used, fly spot counts can be transformed to spots per cm^2^ of card area for comparative analysis (e.g., Gerry et al. 2011).

Spot cards will provide fly activity counts related to fly density and to other measures of fly activity when placed for 7 d on roof supports (Rutz and Axtell 1979, Lysyk and Axtell 1986), but are perhaps more easily placed on support posts (Geden et al. 1999), feed troughs (Lysyk and Axtell 1985), or other easy-to-reach locations where fly spots are abundant. Spot cards may provide a better index of fly activity than fly ribbons when both are placed for a 7-d monitoring period (Axtell 1970a), perhaps due to failure of fly ribbons near the end of the longer sampling period as a result of dust or fly accumulation on the ribbons. In ventilated animal housing, spot cards counts will be higher on the downwind side of building support posts relative to the upwind side (Geden et al. 1999); therefore, consistency of spot card placement during each sampling period is especially important. Place spot cards where birds or machinery will not damage them. Cards can be pinned in place or a small binder clip can be attached to the placement site for easy removal and replacement of spot cards. Face the unlined side of the index card outward for flies to land on.

Using spot cards, house fly activity is recorded as spots per card per week with spot counts serving as an index for overall activity of all pest fly species present at the facility. While the proportion of spots from each fly species may be estimated by the relative density of each fly species recorded using another fly monitoring method (e.g., fly ribbons) (Lysyk and Axtell 1986), this has not been tested and it is best to simply record activity counts from fly spots as ‘overall fly activity’. Suggested action thresholds for spot cards placed on roof supports in egg-layer houses are 50–100 spots per card per week using the standard 3 × 5 in spot card (Axtell 1970a,b; Axtell 1986; Lysyk and Moon 1994). To detect a doubling of fly activity at the lowest action threshold, a minimum of six or seven spot cards are needed (per narrow house or high-rise house, respectively) (Lysyk and Axtell 1986).

Perhaps the biggest challenge to using spot cards as a routine fly monitoring tool is the time that must be committed to counting the hundreds to thousands of fly spots on each card at the end of the sampling period. However, an automated spot card counting algorithm (FlySpotter) has been developed to count the number of spots on a scanned image of a spot card (Gerry et al. 2011). The FlySpotter program is available here: https://www.veterinaryentomology.org/flyspotter-house-fly-monitoring).

### Baited Jug Traps

This trap is a DIY baited fly trap constructed using a translucent plastic 3.8-liter (1 G) milk jug with 5-cm-diameter holes cut into each side into which 25 g of a dry insecticidal fly bait is added (Burg and Axtell 1984, Lysyk and Axtell 1985). Flies attracted to the bait, enter the trap to feed on the bait, and then die within the trap. Each week, dead flies are removed and counted to record a measure of fly activity. Traps baited with commercially available house fly bait will capture house flies almost exclusively, so identification of captured flies is typically not needed (Lysyk and Axtell 1985). Essentially any device baited with insecticidal fly bait can be used in lieu of the baited jug trap (e.g., Geden 2005, Gerry et al. 2011) but there is little published information on other trap designs.

Baited jug traps are commonly placed near the roof peak in narrow poultry houses, at the level of the highest poultry cages in multilevel poultry houses, or about 1 m above accumulated feces in high-rise (deep-pit) poultry houses (Rutz and Axtell 1979, 1981; Burg and Axtell 1984; Lysyk and Axtell 1985; Stafford et al. 1988). A monitoring period of 7 d is typical and these traps are generally not sensitive to dust making them the most effective monitoring device for facilities with dusty conditions such as broiler-breeder houses (Rutz and Axtell 1981). Attractant-baited trap counts are reported to vary along the length of a poultry house (Willson and Mulla 1975, Burg and Axtell 1984) and by proximity to natural attractants or fly development sites (Pickens et al. 1967, Pickens and Miller 1987). It is therefore best to distribute baited jug traps along the long axis of a poultry house. Also, use only translucent white milk jugs to construct the trap since fly capture will vary with different trap colors (Burg and Axtell 1984).

House fly activity is recorded as flies per trap per week. A suggested action threshold for the baited jug trap in caged-layer or broiler-breeder poultry houses is 300–350 flies per trap per week (Rutz 1981, Axtell 1986, Lysyk and Moon 1994). To detect a doubling of fly activity at the lowest action threshold, a minimum of five or six baited jug traps are needed (per narrow house or high-rise house, respectively) though only two baited jug traps are needed for a broiler-breeder house (Lysyk and Axtell 1985, 1986). These minimum trap numbers are similar to the 2–8 traps recommended by Rutz (1981).

While baited jug traps would seem a good method for monitoring house fly activity since flies are captured and easily identified without the sticky mess of fly ribbons, this method is not recommended for long-term house fly monitoring since house flies can rapidly become resistant to insecticides present in baits (Kaufman et al. 2010, Hubbard and Gerry 2020) and any increase in insecticide resistance will prevent direct comparison of fly activity estimates from pre- and post-resistance monitoring periods.

## Cattle Facilities

Methods for monitoring house fly activity have been reported for dairy barns (Pickens et al. 1972, Pickens and Miller 1987), drylot dairies (Gerry et al. 2011), and cattle feedlots (Urech et al. 2004).

### Fly Ribbons

Sticky fly ribbons may be used in enclosed barns, but are not suitable for open facilities including drylots or feedlots where exposure to wind, rain, and dust makes them ineffective (Anderson and Poorbaugh 1965, Rutz and Axtell 1981, Gerry et al. 2011). When used in barns, ribbons are commonly placed to hang from roof supports (Pickens et al. 1972, Morgan and Pickens 1978). Additional placement and monitoring considerations are similar to those described for poultry facilities above. Morgan and Pickens (1978) suggested that four fly ribbons were sufficient for a 10,000 ft^2^ (929 m^2^) barn, and that 50–75 flies per tape were indicative of a ‘moderately heavy’ fly activity level. On a drylot dairy, Gerry et al. (2011) determined that six fly ribbons were needed to detect a doubling of fly activity using the highest weekly mean fly ribbon count (217 flies per tape) as a surrogate for an unknown action threshold. However, Gerry et al. (2011) do not recommend fly ribbons for monitoring fly activity since many fly ribbons were lost to high wind or dust accumulation.

### Sticky Fly Traps

These traps generally have a rigid plastic structure coated with a sticky material to capture flies that land on them or encounter them during flight. There are many commercially available designs for sticky fly traps. Like fly ribbons, sticky fly traps are disposable devices (or at least have a disposable sticky wrap) but their sturdier design makes them more suitable for use in outdoor locations like commercial dairies or cattle feedlots. However, they are considerably more expensive relative to fly ribbons so are recommended only for locations where fly ribbons cannot withstand the environmental conditions.

The placement and use of sticky traps varies with the many trap designs available. Many of these traps are staked to the ground rather than hung from structural supports like fly ribbons. One of the more commonly used sticky traps for monitoring fly activity is the Alsynite biting fly trap (Williams 1973, Broce 1988). Although the Alsynite biting fly trap is commercially marketed to capture stable flies (*Stomoxys calcitrans* (L.)) (Diptera: Muscidae) associated with cattle or horse facilities, it has also been used to monitor house fly activity at cattle facilities (Geden 2005; Gerry et al. 2011; Urech et al. 2004, 2012). On cattle facilities, dust accumulation on these traps may require a more rapid trap replacement schedule (Kaufman et al. 2001, Gerry et al. 2011). Removal of captured flies from sticky fly traps by birds can also be problematic (Gerry et al. 2011) and there are numerous anecdotal reports of small birds captured on sticky fly traps raising concern over use of these traps. Like fly ribbons, sticky traps will capture other fly species so that several pest fly species can be monitored simultaneously. There are no published action thresholds or calculated minimum number of traps required to detect a doubling of fly activity for any sticky traps.

### Baited Traps

With the more challenging outdoor conditions typical of most cattle facilities, traps baited with insecticide are expected to outperform fly ribbons and sticky traps for a 7-d sampling period. Baited jug traps, as described in the section for poultry, or any similar traps containing insecticidal fly bait may be used on cattle facilities (e.g., Geden 2005, Gerry et al. 2011). In cattle barns or other indoor locations, baited jug traps should be placed as described for poultry houses above. In outdoors locations, traps may be distributed across the facility with traps placed at locations away from concentrations of fly-attractive material (manure, feed, silage) that will impact the fly capture rate of the traps (Pickens et al. 1967, Pickens and Miller 1987). There are also a large number of commercially available odor-baited traps for flies (‘stinky fly traps’), but these are a challenge for monitoring fly activity since most capture flies in an stinky liquid within the trap (see review by Gerry 2020). On drylot daries, Gerry et al. (2011) determined that six insecticide-baited traps (buckets with fly bait) were needed to detect a doubling of fly activity using the highest weekly mean trap count (2,760 flies per trap) as a surrogate for an unknown action threshold.

### Spot Cards

On drylot dairies, Gerry et al. (2011) placed 4 × 6 in (10.16 × 15.24 cm) spot cards on support poles within covered feed lanes at a height of 1.8 m above ground to protect spot cards from precipitation and to prevent damage from cattle or machinery. Though any location where fly spots are noted and which is similarly protected from precipitation, cattle, and human activity may be suitable. Gerry et al. (2011) determined that five spot cards were sufficient to detect a doubling of house fly activity using the highest weekly mean spot card count recorded (3,485 spots per card on the 4 × 6 in cards) as a surrogate for an unknown action threshold.

## Other Animal Facilities

There are few published studies where house fly activity was monitored on animal facilities other than poultry or cattle. In swine facilities, spot cards and sticky cards placed ~2 m above swine pens for a 7-d sampling period provided fly activity estimates that were well correlated, suggesting that both methods were similarly effective as a measure of fly activity (Machtinger and Burgess 2020). At small equine farms, house fly activity was recorded at locations where flies were noted to aggregate by using paired sticky traps (Alsyinte trap) and commercial odor-baited traps (Machtinger et al. 2016). The odor-baited traps were emptied of their stinky liquid and captured flies in the field to record fly activity counts by fly species. The relationship of fly capture by these two trap methods was not evaluated. Nevertheless, knowledge gained from these studies along with information on use and placement of monitoring devices in poultry and cattle may be useful for developing a fly monitoring program in other animal facilities.

## Conclusions

Considering the purpose to monitor house flies is to reduce the negative impacts of these flies, monitoring methods that reliably provide an index of house fly activity are most appropriate. Regardless of the monitoring method selected, once suitable locations have been identified for placing monitoring devices these locations must remain unchanged as variation in fly activity estimate is expected according to specific location even within the same animal housing structure.

Monitoring of fly activity should be performed each week, though fly ribbons and sticky traps may be deployed for just a few days each week where dust or fly density prevents a full 7-d sampling period. For routine house fly monitoring, methods that capture flies over at least several days will provide a more reliable estimate of house fly activity than will methods that record fly activity at a single moment in time (‘instantaneous counts’) or over a period of <24 h and which are more impacted by diurnal variation in house fly activity related to environmental conditions (Lysyk and Moon 1994, Gerry 2020).

The action thresholds and number of monitoring devices indicated in each section above are provided only as guidelines for initiating a fly development program. These values will vary among facilities, based on differences associated with facility design, distribution of flies, monitoring method used, and placement of monitoring devices among many other factors. Facility managers can determine an appropriate action threshold for their facility by using an empirical approach to compare facility-specific fly activity data with incidence of negative impacts such as nuisance. In addition, fly activity data recorded during the peak fly season can be used to calculate the precision of monitoring devices and the number of monitoring devices needed to detect a doubling of fly activity near the action threshold (Karandinos 1976, Lysyk and Axtell 1986, Gerry 2020).

### Overall Recommendations

Spot cards are inexpensive devices that are a suitable for monitoring fly activity on most animal facilities at locations where fly spots accumulate and where spot cards will remain dry and protected from damage by animals and machinery. Spot cards are simple to install and do not require handling of sticky traps, foul-smelling attractants, or insecticides. Also, counting and recording fly spots does not require insect identification skill. Spot cards are less impacted by dust or other environmental conditions than are other monitoring devices and can typically remain in place for a full 7-d monitoring period so that activity estimates represent the full range of environmental conditions during the week (Lysyk and Moon 1994). Spot cards can be retained for years as a record of past fly activity. While counting fly spots on each spot card is tedious, availability of a program to count spots on scanned images of each card will greatly reduce this time-consuming aspect of using spot cards (Gerry et al. 2011).

Spot cards do have some limitations, however. Where multiple fly species are abundant, the relative activity of each species cannot be distinguished since spots on the cards cannot be identified to species. Instead, at these facilities spot cards provide a measure of overall fly activity (Axtell 1970a). While other fly monitoring techniques (e.g., fly ribbons) may be used in conjunction with spot cards to determine the relative prevalence of each fly species, it is not clear that the relative contribution of any fly species to the total fly spot count is related to the actual prevalence of each fly species at the facility. Additionally, each fly may deposit more than one spot, and if the spot deposition rate varies with temperature or other environmental conditions, then fly activity measured by spot cards may be inflated under some conditions relative to devices that record each fly only once (e.g., traps).

Fly ribbons are also a low-cost method for monitoring house fly activity and have been successfully used indoors, particularly in poultry housing. Fly ribbons are recommended over spot cards for animal facilities where several pest fly species are abundant and it is important to distinguish fly activity for each species separately (Axtell 1970a, Lysyk and Axtell 1986). Fly ribbons are not recommended for use outdoors or in other locations where dust, direct sunlight, or high winds are expected. Sticky traps or sticky cards may be used in place of fly ribbons where a sturdier trap is needed or for ease of trap placement; however, there is little published information to provide guidance with placement of these devices or interpretation of fly activity counts. Dust or high fly density may require that fly ribbons or sticky traps be deployed for less than a full 7-d sampling period (Axtell 1970a). The most appropriate sampling period for fly ribbons and sticky traps can be determined by trial use during the peak fly season. If sticky traps are used outdoors, bird strike can be reduced by positioning chicken wire over the trap leaving a gap of ≥5 cm between the wire barrier and the trap. Sticky fly traps cost considerably more than other monitoring methods, but costs can be reduced over time by selecting sticky fly traps with a disposable outer sticky sleeve so that the body of the trap can be reused.

Outdoors, baited jug traps, or other traps baited with insecticidal fly bait can provide fly activity monitoring over a full 7-d sampling period since these traps are less impacted by dust or other environmental conditions. Overall, these traps are easy-to-use and are relatively inexpensive as compared to sticky traps that might also be used outdoors. However, house flies can rapidly develop resistance to insecticides in fly baits (e.g., Kaufman et al. 2010, Hubbard and Gerry 2020), and house fly resistance to the insecticidal bait will alter the fly activity estimate using these traps. While baits to which flies have developed resistance can be replaced with a new bait or a new insecticide, the attractiveness of the new bait material may differ so that fly activity recorded before and after the bait change cannot be directly compared. Nevertheless, insecticide-baited traps may be the only option where spot cards or sticky traps cannot be used due to environmental conditions.

Given advances in video technology and machine vision systems (Liu et al. 2017), pest monitoring systems of the future may offer real-time data without the need to capture flies or count fly spots. For example, video recording of a defined air space or landing target coupled with algorithms to identify insect species could provide fly activity data that are uploaded wirelessly to a database to guide more targeted and more responsive fly control efforts. However, until these methods have been developed and tested, the monitoring methods described above remain the best options for use in an IPM program to manage house flies associated with animal production facilities.
